# Single and Combined Effects of Phenanthrene and Silver Nanoparticles on Denitrification Processes in Coastal Marine Sediments

**DOI:** 10.3390/microorganisms12040745

**Published:** 2024-04-06

**Authors:** Pengfei Sun, Jie Bai, Jie Lian, Yongyu Tan, Xi Chen

**Affiliations:** 1 Key Laboratory of Tropical Marine Ecosystem and Bioresource, Fourth Institute of Oceanography, Ministry of Natural Resources, Beihai 536000, China; sunpengfei@4io.org.cn (P.S.); lianjie@4io.org.cn (J.L.); tanyongyu@4io.org.cn (Y.T.); 2 College of Marine Life Sciences, Ocean University of China, Qingdao 266100, China; 3 Guangxi Beibu Gulf Key Laboratory of Marine Resources, Environment and Sustainable Development, Fourth Institute of Oceanography, Ministry of Natural Resources, Beihai 536000, China; 4 College of Environmental Science and Engineering, Ocean University of China, Qingdao 266100, China; baijie@ouc.edu.cn

**Keywords:** phenanthrene, silver nanoparticles, denitrification, denitrifying bacterial community, coastal marine sediments

## Abstract

The increasing production and utilization of polycyclic aromatic hydrocarbons (PAHs) and commercial silver nanoparticles (AgNPs) have raised concerns about their potential environmental release, with coastal sediments as a substantial sink. To better understanding the effects of these contaminants on denitrification processes in coastal marine sediments, a short-term exposure simulation experiment was conducted. We investigated the effects of single and combined contamination of phenanthrene (Phe) and AgNPs on denitrification processes in a coastal marine sediment. Results showed that all contaminated treatment groups had different degrees of inhibitory effect on denitrification activity, denitrifying enzyme activity, total bacteria count and denitrifying genes. The inhibitory effect sequence of each treatment group was combined treatment > AgNPs treatment > Phe treatment. Moreover, the inhibitory effects of denitrifying genes were much larger than that of total bacteria count, indicating that the pollutants had specific toxic effects on denitrifying bacteria. The sequence of sensitivity of three reduction process to pollutants was N_2_O > NO_2_^−^ > NO_3_^−^. All contaminated treatment groups could increase NO_3_^−^, NO_2_^−^ and N_2_O accumulation. Furthermore, according to the linear relationship between functional gene or reductase and denitrification process, we also found that the abundance of denitrifying genes could better predict the influence of Phe and AgNPs on sediment denitrification than the denitrifying bacterial diversity. In addition, at the genus level, the community structure of *nirS*- and *nosZ*-type denitrifying bacteria changed dramatically, while changes at the phylum level were comparatively less pronounced. Single and combined contamination of Phe and AgNPs could reduce the dominance of *Pseudomonas*, which may lead to a potential slow-down in the degradation of Phe and inhibition of denitrification, especially the combined contamination. Overall, our study revealed that combined contamination of Phe and AgNPs could lead to an increase in NO_3_^−^, NO_2_^−^ and N_2_O accumulation in coastal sediment, which poses a risk of eutrophication in coastal areas, exacerbates the greenhouse effect and has adverse effects on global climate change.

## 1. Introduction

Nitrogen (N) is a crucial and indispensable element for organisms and ecosystems [1]. Excessive use of nitrogenous fertilizers can lead to not only an overabundance in nitrogen discharge but also eutrophication, resulting in the deterioration of water quality, decreased biodiversity and destruction of aquatic habitats [2]. In the coastal ecosystem, denitrification is a significant process, where bacteria transform nitrate into nitrite and then to nitrogen gas and other gaseous forms of nitrogen, playing a vital role in controlling the fate of nitrogen in this environment [3]. Functional genes such as *amoA* (encode ammonia onooxygenase), *nxrA* (nitrite oxidase), *narG*/*napA* (nitrate reductase), *nirK*/*nirS* (nitrite reductase), *norB* (nitric oxide reductase) and *nosZ* (nitrous oxide reductase) primarily drive this multi-step process [4]. During denitrification, an intermediate gas, N_2_O, impacts the atmosphere by acting as a potent ozone-depleting substance and a greenhouse gas [5,6]. It is crucial to note that the global warming potential of N_2_O is 310 times higher than carbon dioxide [3].

Polycyclic aromatic hydrocarbons (PAHs) belong to a group of persistent organic pollutants (POPs) and are known for their toxicity, which stems from their potential hazardous properties, recalcitrance and widespread occurrence. These compounds are generated through both natural processes and human activities, leading to their wide distribution in the environment. The PAHs content in the sediments of different types of water varies, and the PAHs content of sediments in ports, bays and estuaries is usually higher [7].

Silver nanoparticles (AgNPs) are becoming increasingly important nanomaterials with broad applications in both traditional industries and emerging fields, primarily due to their unique antibacterial properties. It was estimated that global consumption of AgNPs will exceed 360 tons per year [8,9]. Some researchers predicted the growth rate of AgNPs concentration in various types of sediments in Europe, the United States and Switzerland by using a probabilistic model, and the ranges of the growth rates were 978–8593 ∆ng kg^−1^y^−1^, 153–1638 ∆ng kg^−1^y^−1^ and 965–10184 ∆ng kg^−1^y^−1^, respectively [10]. With the extensive utilization of AgNPs, a considerable amount of AgNPs infiltrates rivers through various pathways, eventually accumulating in estuarine areas. Then, the majority of AgNPs tend to settle into sediment, while less than 5% persist in seawater [11]. With the increase in the production and emission of PAHs and AgNPs products, the presence of PAHs and AgNPs in coastal areas poses a substantial threat to the environment and aquatic organisms due to their persistent and bio-accumulative nature in the environment [10,12,13].

Recent studies have focused on the impacts of PAHs and AgNPs on denitrifying bacteria and denitrification processes in the soil and sediment. These studies suggested that exposure to PAHs and AgNPs, discretely, not only reduced the abundance and diversity of denitrifying genes (such as *narG*, *nirK*, *nirS*, *nrfA*, *nosZ* and so on) in soil and sediments but also lowered the potential denitrification activity (PDA), further impacting the community structure and function of denitrifying bacteria [8,9,14,15,16]. As a consequence, the incomplete denitrification can inhibit inorganic nitrogen decomposition and cause large N_2_O emission, which might lead to eutrophication, global warming and stratospheric ozone destruction [17]. Jin et al. (2022) [18] revealed that a mixture of PAHs promoted PAHs functional genes while inhibiting denitrification functional genes. Additionally, some researchers indicated that AgNPs have adverse effects on the related bacteria and functional genes [19,20]. For instance, the addition of 10 mg/L AgNPs could cause an inhibitory effect on the abundance of functional genes such as *nirK* and *nirS*, as well as the predominant bacteria Paracoccus, causing a halt in the release rate of N_2_O and N_2_, thereby increasing the accumulation of inorganic nitrogen [21]. AgNPs, to a large extent, could also decrease the NO_3_^−^ reduction rate in the soil. The degree of inhibition is closely related to the concentration of silver nanoparticles added [22]. Moreover, the presence of AgNPs could inhibit the activity of nitric oxide reductase (NOR) and nitrous oxide reductase (NOS) and the abundance of corresponding encoding genes of denitrification process, *norB* and *nosZ* genes, in the sediments of hypertrophic and mesotrophic lakes [23]. A single exposure to 10 mg/L of AgNPs has been proven to cause an obvious toxicity on the activity and abundance of denitrifying bacteria in the first 30 days in sediments of the Taihu Lake, China. The toxicity was reflected by the decrease in nicotinamide adenine dinucleotide (NADH) formation, electron transport system (ETS) activity, NO_2_^−^ reductase activity (NIR), N_2_O reductase activity (NOS) and *nirK* gene copy number, ultimately leading to a significant decline in denitrification rate [24]. However, the above studies were all focused on single effects of PAHs or AgNPs on the denitrification processes in the soil or sediment. Previous studies have shown that, in addition to their inherent toxicity, nanomaterials in the environment can also interact with different types of pollutants, thereby affecting their biological effects in the environment [12]. Dai et al. (2013) [25] showed that the combined contamination of multi-wall carbon nanotubes and nano-zinc oxide with phenanthrene (Phe), a PAH, could induce the generation of free radicals in the brain and liver tissues of crucian and increase the enrichment of Phe in the brain, eye and embryo of fish. Schwab et al. (2013) [26] found that carbon nanomaterials can adsorb the herbicide diuron, and their combined exposure can enhance the toxic effect of single contamination on *Chlorella vulgaris*. The combined exposure of nano-microplastics and PAHs could also increase the toxicity effects on *Chlamydomonas reinhardtii* via cell membrane damage and modifications in the physiological system. We have already explored the acute (24 h) toxicity effects of PAHs or AgNPs exposure on the denitrification processes in estuarine and marine sediments [27,28]. However, research on their combined effects on denitrification remains limited. Given the widespread presence of PAHs and engineered AgNPs in the aquatic system, it is critical to investigate their combined effects on denitrification.

Pollutants tend to be more concentrated and complex in coastal areas. Nanomaterials and co-pollutants form new complexes through physical or chemical interactions, and the differences in morphological properties can change the toxic effects of nanomaterials and co-pollutants [29]. The coastal marine sediment of Jiaozhou Bay (JZB), where several rivers meet the sea, was selected as the sampling site. Situated in northern China (35°43′–36°18′ N, 120°04′–120°23′ E), the JZB is home to Qingdao Port and has experienced significant alterations in the ecosystem due to human activities, including bank re-alignment, port construction, shipyard establishment and the development of fishing harbors. Moreover, the ecosystem of the JZB is influenced by inputs from agricultural runoff, as well as urban and industrial sewage, which contribute nutrients and waste from residential, industrial and agricultural areas, further impacting its environmental health. Therefore, the JZB is also a main gathering area of AgNPs. Meanwhile, as a typical representative of three-ringed PAHs, the detection rate of Phe in the JZB and the East China Sea and other sea areas has increased in recent years [30,31]. Herein, the JZB was selected as the coastal area, while Phe and AgNPs represented PAHs and nanomaterials, respectively. Through short-term exposure simulation experiments, this study aimed to evaluate the response of denitrifying capacity, the denitrification enzyme activity, the abundance of denitrifying genes and the community structure and diversity of denitrifying bacteria when exposed to single and combined contamination of Phe and AgNPs in a typical coastal marine sediment. In addition, the inhibition mechanism of Phe and AgNPs on denitrification processes and the denitrifying genes were also discussed. This study provides valuable insights into the effects of single and combined contamination of Phe and AgNPs on denitrification under more environmentally relevant conditions.

## 2. Materials and Methods

### 2.1. Sample Collection

**Figure 1:**
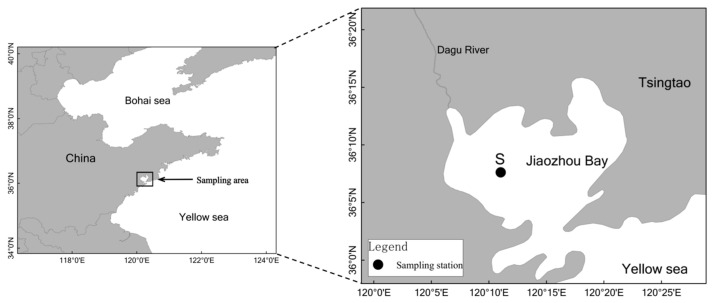
The diagram of the sampling station in this study.

Surface sediment (0–5 cm) samples were collected from the station S (36°07′37″ N, 120°11′02″ E) in July 2017 (Figure 1). To prevent sediment oxidation, the sediment was carefully sealed in polyethylene bags, placed in covered buckets and stored at 4 °C in a dark, cold room without freezing until further use. Before conducting sediment characterization and the following experiments, the sediment was further sieved thoroughly to remove debris larger than 1 mm [32]. Seawater samples were collected from the water column above the sediment using the water sampler. The collected seawater was stored in bottles (polyethylene acid) cleaned with 10% HCl solution and rinsed with milli-Q water. Seawater samples were immediately filtered using 0.22 μm membrane filters, then stored at 4 °C. The contents of PAHs and Ag in the sediment was determined by gas chromatography-mass spectrometry and inductively coupled plasma mass spectrometry, respectively. The sediment’s PAHs content was found to be 0.041 ± 0.004 mg/Kg, while the Ag content was 0.019 ± 0.002 mg/Kg. The background values of PAHs and Ag in the sediment are negligible compared to the experimental addition values.

### 2.2. Experiment Setups

#### 2.2.1. Exposure to Phe and AgNPs

The exposure concentration of Phe and AgNPs in this study was determined based on the 24 h EC_50_ (concentration of Phe or AgNPs in sediment causing 50% inhibition of PDA) of the pollutant acting on sediment PDA alone. The EC_50_ of phenanthine and nano-silver acting on sediment PDA in Jiaozhou Bay was 61.19 mg kg^−1^ and 147.47 mg kg^−1^, respectively, through the 24 h laboratory simulated culture experiment [27,28].

**Figure 2:**
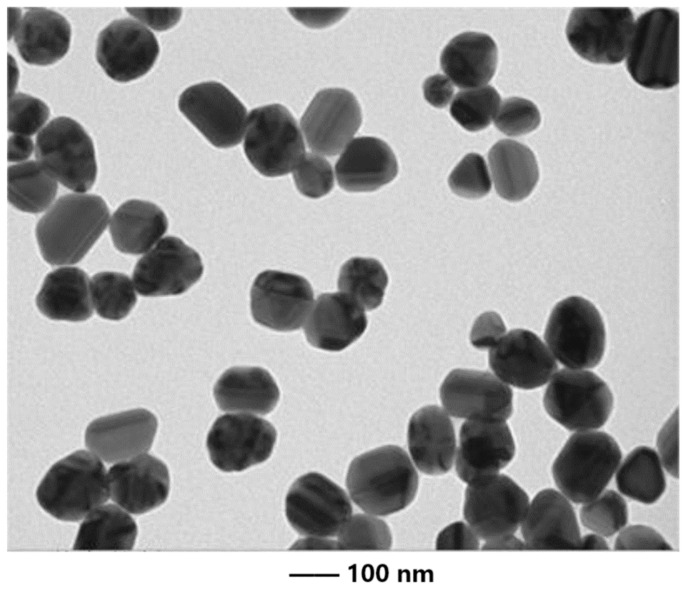
Scanning electron microscopy picture of silver nanoparticles.

Before the experiment, crystalline Phe (L01921, Alfa Aesar, Lancaster, PA, USA) was dissolved in acetone and uniformly sprayed onto dry sediment, resulting in sub-samples with Phe levels of 0 (Phe-free) and 3059.5 mg kg^−1^ of dry sediment weight. The sub-samples were mixed thoroughly and left overnight in the dark to allow the solvent to evaporate. Meanwhile, seawater was amended with 50 mM KNO_3_ and 1% glucose, respectively. The AgNPs (CAS No. 7440-22-4), purchased from Sigma-Aldrich (St. Louis, MO, USA), had an average particle diameter of 100 nm (Figure 2). The original stock suspension of AgNPs were prepared by dispersing them in the aforementioned seawater to make a concentration of 147.47 mg L^−1^. The suspension then underwent ultrasonication for 1 h (20 kHz and 500 W) at 4 °C to break down aggregates before being diluted to exposure concentrations.

#### 2.2.2. Six Days Batch Exposure Experiment

To evaluate the single and combined effects of Phe and AgNPs on denitrification, a 6-day denitrification treatment was conducted in 150 mL serum bottles. The exposure experiments were divided into four treatment groups: (1) SC (sediment without any Phe and AgNPs); (2) SP (sediment contaminated with Phe (61.19 mg kg^−1^ wet sediment)); (3) SA (sediment contaminated with AgNPs (147.47 mg kg^−1^ wet sediment)); (4) SJ (sediment contaminated with Phe (61.19 mg kg^−1^ wet sediment) and AgNPs (147.47 mg kg^−1^ wet sediment)). Three replicates were prepared for each group. The treatment process of each experimental group is as follows: For the SC and SP treatment groups, 1 g of 0 and 3059.5 mg kg^−1^ dry weight sediments were mixed with 50 g wet sediment and 50 mL filtered seawater amended with 50 mM KNO_3_ and 1% glucose in a 150 mL serum bottle. For the SA treatment group, 1 g of 3059.5 mg kg^−1^ dry sediment was mixed with 50 g wet sediment and 50 mL AgNPs stock suspensions in a 150 mL serum bottle. For the SJ treatment group, 1 g of 3059.5 mg kg^−1^ dry weight sediment was mixed with 50 g wet sediment and 50 mL AgNPs stock suspensions in a 150 mL serum bottle. The stock suspension was sonicated for 30 min at 4 °C before use. All serum bottles were hermetically sealed with a butyl stopper and aluminum crimp, then purged with N_2_ for 15 min to remove O_2_. The bottles were incubated at a constant temperature of 25 °C with continuous shaking at 80 rpm in the dark for 6 days. After 6 days of exposure, all bottles were shaken to homogenize the samples, then 5 mL sediment slurries were collected in 10 mL sterilized centrifuge tube for DNA extraction. Additionally, 5 mL sediment slurries were mixed with 0.2 mL of 1 g L^−1^ HgCl and collected in 10 mL sterilized centrifuge tubes, then centrifuged with 0.22 μm filter to measure the content of NO_3_^−^ and NO_2_^−^. Six 5 mL sediment slurries were taken from each parallel sample for subsequent denitrification activity culture experiments and determination of denitrifying reductase activity. Furthermore, 2 g wet sediment was transferred to the sterilized centrifuge tubes containing 5 mL of saline solution (0.22 μm filtered, 9 g L^−1^ NaCl, 200 mL of 0.22 μm filtered, 12.5% *v/v* Tween 80) and fixed with 100 μL of formaldehyde (0.22 μm filtered, 4% *v/v*) for total cell count analysis using flow cytometry.

#### 2.2.3. Denitrification Activity

The denitrification rate was measured by the acetylene (C_2_H_2_) inhibition technique described by Sørensen (1978) [33]. Six 5 mL pre-incubated sediment slurries from each replicate of all the above-mentioned treatment groups were collected by centrifuging at 1500 rpm and washed thrice with clean seawater to remove any potential NO3^−^ residue. These sediment slurries were then shaken and incubated at room temperature overnight to ensure uniform microbial activity across all treatment groups. Then, the sediment was transferred into 15 mL serum bottle with 5 mL filtered seawater (amended with 300 mM KNO_3_ and 2 mM glucose). All serum bottles were hermetically sealed with a butyl stopper and aluminum crimp, then purged with N_2_ for 15 min to remove O_2_. Each treatment group was divided into three experimental groups, and three replicates were set for each experimental group. The first groups were filled with acetylene (C_2_H_2_, 20%, *v/v*) to prevent N_2_O from being further reduced to N_2_. Gas samples (6 mL) were collected from each serum bottle of the second groups after vigorous shaking, then injected into 10 mL vacuum bottles for later N_2_O analysis at time zero. All serum bottles of the first and third groups were shacked at 80 rpm in the dark for 4 h at a constant temperature of 25 °C. After 4 h, gas samples (6 mL) were collected from each serum bottle and injected into a 10 mL evacuated serum vials for later N_2_O analysis. Subsequently, the sediment slurries were treated with 0.2 mL of 1 g L^−1^ HgCl and collected in 10 mL sterilized centrifuge tubes, followed by centrifugation and filtration through a 0.22 μm filter for the measurement of NO_3_^−^ and NO_2_^−^.

Denitrification rate and N_2_O accumulation were calculated by quantifying N_2_O produced with and without C_2_H_2_, respectively. N_2_O reduction via denitrification was determined as the difference between the N_2_O produced with and without C_2_H_2_ [34]. The NO_3_^−^ reduction rate was calculated as the difference between concentrations of N compounds measured at *T*_0_ and *T*_4h_. Similarly, the NO_2_^−^ reduction rate was calculated by subtracting the NO_3_^−^ reduction from the NO_2_^−^ residues.

#### 2.2.4. Determination of Denitrifying Reductase

The measurements of key enzyme activities can provide insights into the potential influences of contaminations [35,36]. Six 5 mL pre-incubated sediment slurries from each replicate of all the above-mentioned treatments were transferred to 10 mL sterile centrifuge tube. Cells were harvested by centrifugation (12,000 rpm for 10 min), washed thrice with 0.1 M phosphate-buffered saline (PBS) (pH 7.4) to eliminate any possible NO_3_^−^ residue and resuspended in the same buffer at 4 °C. Crude cell extracts were prepared by sonication of cells for 1 min at 4 °C and 20 kHz, followed by centrifugation at 12,000 rpm for 10 min at 4 °C, then the extracts were immediately used for determination of enzyme activities.

NO_3_^−^ and NO_2_^−^ reductase (NAR and NIR) activity: 2 mL of mixed culture medium was added to an anaerobic bottle. The mixed culture medium contained 10 mM PBS, 10 mM methyl viologen, 5 mm Na_2_S_2_O_4_ and 1 mM NaNO_3_ or NaNO_2_. Then, 0.1 mL of the above-mentioned supernatant was added to the bottle. The mixture was incubated at room temperature (25 °C) for 30 min under anaerobic conditions, and the NAR and NIR activity were calculated through the reduction of NO_3_^−^ and NO_2_^−^ [37,38].

The measurement of nitrous oxide reductase (N_2_OR) activity was based on the methyl viologen method [39], and the improved process was as follows: 1.5 mL of 20 mM Tris HCl buffer (pH 7.0), 0.2 mL of 10 mM methyl viologen and 0.1 mL of the above-mentioned supernatant were added to the 3.5 mL anaerobic cuvette, which was filled with nitrogen. A total of 50 μL of 50 mM Na_2_S_2_O_4_ was injected into the anaerobic cuvette by a micro syringe, which can provide reducing electrons to methyl viologen and stabilize the OD600 of the mixed reaction solution at 1.0–1.2. Then, a micro syringe was used to inject 0.1 mL of N_2_O-saturated solution into the anaerobic cuvette, and the cuvette was cultured in the ultraviolet spectrophotometer at room temperature (25 °C). In addition, the fluorescence value was measured at the same time. The change curve of OD600 was drawn to calculate the average slope, which indicates the N_2_OR activity (N_2_OR activity (U) = average slope/molar extinction coefficient of substrate) The molar extinction coefficient of substrate methyl viologen was 5.8 mM in this study.

### 2.3. Total Bacterial Count

The subsamples of slurries collected were treated as follows: The samples were stirred at 150 rpm for 15 min to ensure proper mixing and then subjected to sonication for 30 s at a low intensity (0.5 cycle, 20% amplitude). The sonication process was performed to disrupt any cell aggregates and ensure a more accurate analysis of bacterial cells in the cleaned suspensions. The cleaned bacterial cells in the suspension were subsequently analyzed by a BD Accuri C6 flow cytometer (Becton, Dickinson and Company, Franklin Lakes, NJ, USA) [27,40].

### 2.4. DNA Extraction and Quantitative Real-Time Polymerase Chain Reaction (RT-qPCR)

The genomic DNA was extracted from 1 g wet weight of sediment using a Fast DNA SPIN kit for soil (SK8233, Sangon Biotech Co., Ltd., Shanghai, China) following the manufacturer’s instructions and stored at −80 °C for later use in RT-qPCR.

The *nirG*, *nirS* and *nosZ* genes were selected for denitrifying bacteria. The primer pairs 1960m2F and 2050m2R-GC [39], Cd3aF and R3cdR-GC [41] and F2 and R2 [42] were used to amplify *narG*, *nirS* and *nosZ* gene fragments, respectively. The product length of the three genes were 110, 406 and 267 bp, respectively. Quantification of denitrifying genes was performed by a Roche LightCycler 480 (Roche, Basel, Switzerland). The 25 μL reaction mixtures contained 10 μL of SYBR Green Fast qPCR Master Mix (Roche, Basel, Switzerland), 0.4 μL of each 20 mM primer, 7.2 μL ddH_2_0 and 2 μL of template DNA. Thermal cycling conditions for *nirS* and *nosZ* genes were as follows: pre-incubation at 95 °C for 3 min, 45 cycles consisting of denaturation at 94 °C for 7 s, annealing at 57 °C for 10 s and extension at 72 °C for 30 s. After the cycling, a melting curve analysis was conducted from 60–95 °C with a heating rate of 0.11 °C s^−1^ and continuous fluorescence measurement. Finally, the samples were cooled down to 40 °C. The gene expression measurement was performed by RT-qPCR in triplicate, and the mean value was used for final analysis. The relative abundance of each gene was analyzed by the 2^−△△CT^ method [43]. To ensure accuracy, all qPCR reactions, including the unknown samples, were performed in triplicate. In order to account for any contamination or background noise, no-template control (NTC) treatments were included in all experimental runs.

### 2.5. Gene Amplification, High Throughput Sequencing and Data Analysis

The V4 region of the bacterial 16S rRNA gene (about 290 nucleotides) was targeted with the primer pairs of 515f and barcoded 806r [44]. Polymerase chain reaction (PCR) was performed to amplify the genes, including *nirS* and *nosZ*. The primers used for gene detection were those mentioned above. All samples were amplified in triplicate, and no-template controls were included at all steps of the process. Triplicate PCR amplicons were pooled together. The PCR reactions were carried out in 30 μL reactions with 15 μL of Phusion^®^ High-Fidelity PCR Master Mix (New England Biolabs Inc., Ipswich, MA, USA), 3 μL of each primer (2 μM), 10 μL of template DNA (5–10 ng) and 2 μL sterilized water. Thermal cycling consisted of initial denaturation at 98 °C for 1 min, followed by 30 cycles of denaturation at 98 °C for 10 s, annealing at 50 °C for 30 s, elongation at 72 °C for 30 s and a final step at 72 °C for 5 min. The same volume of 1× loading buffer (containing SYB green) with PCR products were mixed and electrophoresis was conducted on 2% agarose gel for detection. PCR products with a bright band were mixed in equal density ratios and purified with GeneJET Gel Extraction Kit (Thermo Scientific, Waltham, MA, USA). The purified PCR amplicons products were sequenced on the Illumina MiSeq platform (Illumina Inc., San Diego, CA, USA) at the Novogene Bioinformatics Technology Co., Ltd. (Beijing, China).

The paired-end reads from the original DNA fragments were merged by using FLASH (Version 1.2.7) [45], and UPARSE (Version 7.1), a chimera filtering approach, was employed to pick operational taxonomic units (OTUs) with 97% similarity [46] on the Bio-Linux platform. Representative sequences from each OTU were analyzed using the QIIME (Version 1.7.0) [47] software package (Quantitative Insights into Microbial Ecology), and in-house Perl scripts were used to analyze alpha (within samples) and beta (among samples) diversity analysis. We picked a representative sequence for each OTU and used the RDP classifier [48] to annotate taxonomic information for each representative sequence. The local nucleotide databases of the denitrifying genes were created by downloading DNA sequences of *nirS* and *nosZ* genes from NCBI (http://www.ncbi.nlm.nih.gov/. Accessed on 6 June 2023).

Alpha diversity was computed by rarifying the OTU table, and three metrics were calculated: Chao1, which estimates the species abundance, Observed Species, which estimates the amount of unique OTUs found in each sample and the Shannon index. Rarefaction curves were generated based on these three metrics. QIIME calculates both weighted and unweighted UniFrac, which are phylogenetic measures of beta diversity. Beta diversity was assessed using unweighted UniFrac for Principal Coordinate Analysis (PCoA) and Unweighted Pair Group Method with Arithmetic mean (UPGMA) Clustering. PCoA helps visualize complex, multidimensional data by transforming a distance matrix into a new set of orthogonal axes, demonstrating maximum variation by the first principal coordinate, second maximum by the second principal coordinate and so on. UPGMA Clustering, a hierarchical clustering method, uses average linkage to interpret the distance matrix. To further analyze the microbial diversity differences between the samples, several statistical analysis methods were applied, including *T*-test, MetaStat, LEfSe, Anosim and MRPP.

## 3. Results and Discussion

### 3.1. Effect of Single and Combined Contamination of Phe and AgNPs on Denitrification Process

The denitrification process consists of four main steps: nitrate reduction, nitrite reduction, nitric oxide reduction and nitrous oxide reduction. While the reduction rates of NO_3_^−^, NO_2_^−^, NO and N_2_O can partially reflect the denitrification ability, the denitrification rate is usually calculated by measuring the N_2_ production [45]. Since directly determining N_2_ can be challenging, many researchers employ the acetylene inhibition technique to hinder the reduction of N_2_O to N_2_ and then calculate the denitrification rate using N_2_O production as a proxy for N_2_ production [14,49,50]. In this paper, we used the Potential Denitrification Activity (PDA), which is measured through the acetylene inhibition technique, to represent the denitrification rate.

#### 3.1.1. Nitrate and Nitrite Reduction

**Figure 3:**
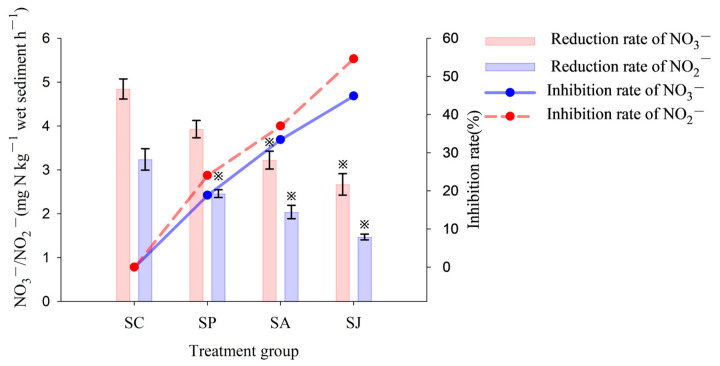
Reduction and inhibition rate of nitrate (NO_3_^−^) and nitrite (NO_2_^−^) of different treatment groups. Note: “※” Represent the significant difference between group SC and the other three groups (ANOVA, *p* < 0.05; the same below).

The control group showed the highest NO_3_^−^ and NO_2_^−^ reduction rates at 4.85 mg N kg^−1^ h^−1^ and 3.24 mg N kg^−1^ h^−1^, respectively (Figure 3). The NO_3_^−^ and NO_2_^−^ reduction rates of the combined contaminated treatment groups were the lowest at 2.67 mg N kg^−1^ h^−1^ and 1.47 mg N kg^−1^ h^−1^, respectively. The reduction rates of NO_3_^−^ and NO_2_^−^ in the Phe, AgNPs and combined contaminated treatment groups were all found to be inhibited to varying degrees compared to the control group. Specifically, the NO_3_^−^ reduction rates in the AgNPs and combined contaminated treatment groups exhibited significant differences from the control group (ANOVA, *p* < 0.05), while the NO_2_^−^ reduction rates in all three contaminated treatment groups were also significantly different from the control group (ANOVA, *p* < 0.05). Moreover, the inhibition rates of NO_3_^−^ and NO_2_^−^ reduction were both highest in the combined contaminated treatment group with 44.89% and 54.63%, respectively, followed by the AgNPs treatment group with 33.4% and 37.04%, respectively, and the Phe treatment group was the lowest with 19.60% and 24.07%, respectively. Therefore, the combined contamination of Phe and AgNPs had a stronger inhibition effect on NO_3_^−^ and NO_2_^−^ reduction than that of single contamination of AgNPs; meanwhile, the inhibition effect of single contamination of Phe was the weakest (ANOVA, *p* < 0.05). In addition, the inhibition effect of all contaminated treatment groups on NO_2_^−^ reduction was stronger than that of NO_3_^−^ reduction rate (ANOVA, *p* < 0.05). The findings indicated that the single and combined contamination of Phe and AgNPs could lead to elevated levels of NO_3_^−^ and NO_2_^−^ accumulation in marine environment, consequently posing an increased risk of eutrophication in the study area.

#### 3.1.2. Potential Denitrification Activity

**Figure 4:**
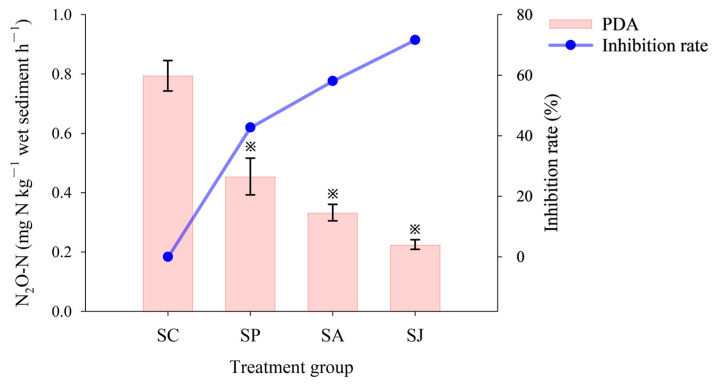
Potential denitrification activity and inhibition rate of different treatment groups. “※” Represent the significant difference between group SC and the other three groups (ANOVA, *p* < 0.05).

In comparison to the control group, the PDA in different contaminated treatment groups was significantly inhibited to varying degrees (ANOVA, *p* < 0.05) (Figure 4). The highest (0.79 mg N kg^−1^ h^−1^) PDA was recorded in the control group, followed by the AgNPs and Phe treatment groups, where the PDA was 0.45 mg N kg^−1^ h^−1^ and 0.33 mg N kg^−1^ h^−1^, with inhibition rates of 58.11% and 42.75%, respectively. The combined contaminated treatment group exhibited the lowest reduction rate, measuring 0.23 mg N kg^−1^ h^−1^, with a notable inhibition rate of 71.66%. Therefore, results indicated that the inhibition effect of the three contaminated treatment groups on PDA varied in the order combined contaminated treatment > AgNPs treatment > Phe treatment, which was consistent with the inhibition effect on NO_3_^−^ and NO_2_^−^ reduction rates, and the inhibition effect on PDA was stronger than that of NO_3_^−^ and NO_2_^−^ reduction rates (ANOVA, *p* < 0.05).

#### 3.1.3. N_2_O Accumulation and Reduction

**Figure 5:**
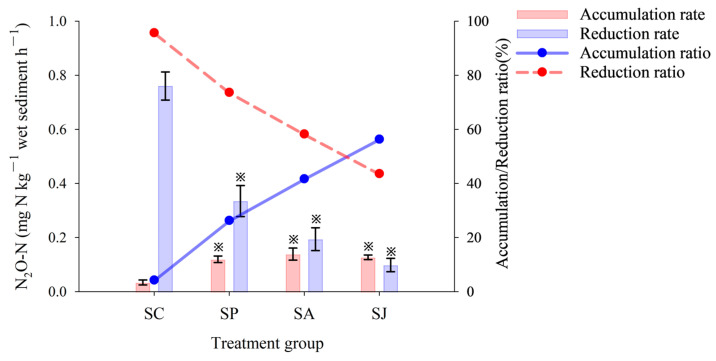
N_2_O accumulation and reduction rate of different treatment groups. “※” Represent the significant difference between group SC and the other three groups (ANOVA, *p* < 0.05).

The change trend of the N_2_O reduction rate was consistent with the PDA, and the control group recorded the highest, in which the PDA was 0.76 mg N kg^−1^ h^−1^, followed by the Phe and AgNPs treatment groups, and the combined contaminated treatment group recorded the lowest, with a PDA of 0.10 mg N kg^−1^ h^−1^ (Figure 5). Moreover, the N_2_O accumulation rate in the control group was the lowest (33.87 ug N kg^−1^ h^−1^), the N_2_O accumulation rates of the three contaminated treatment groups significantly increased compared to the control group (ANOVA, *p* < 0.05) and the N_2_O accumulation rate of the AgNPs treatment group was the highest at 0.14 mg N kg^−1^ h^−1^. The N_2_O accumulation ratios ranged from 4.27% to 56.37%; the N_2_O accumulation ratio was the lowest in the control group, followed by the Phe and AgNPs treatment groups, and was highest in the combined treatment group. However, the change trend of the N_2_O reduction ratio is the opposite of that of the accumulation ratio, and the reduction ratios ranged from 43.63 to 95.73%. The above results imply that single and combined contamination of Phe and AgNPs could lead to the accumulation of N_2_O, an important greenhouse gas known for its stable chemical properties, long retention time in the atmosphere and average atmospheric lifetime of 100–150 years. Furthermore, N_2_O can be transported to the atmospheric stratosphere to deplete ozone through photochemical reactions, which in turn leads to ozone layer destruction [5,51].

### 3.2. Effect of Single and Combined Contamination of Phe and AgNPs on Denitrifying Reductase Activities

**Figure 6:**
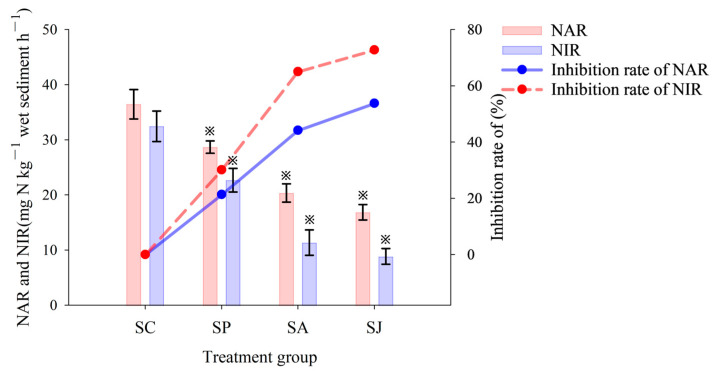
Activity and inhibition rate of nitrate (NAR) and nitrite (NIR) reductase of different treatment groups. “※” Represent the significant difference between group SC and the other three groups (ANOVA, *p* < 0.05).

The short-term effects of single and combined contamination of Phe and AgNPs on NAR and NIR activities of sediments in the JZB are shown in Figure 6. Both NAR and NIR activities in the control group were the highest, at 36.45 mg N kg^−1^ h^−1^ and 32.45 mg N kg^−1^ h^−1^, respectively. This was followed by the Phe and AgNPs treatment groups, where the NAR and NIR activities of the Phe treatment group were 36.45 mg N kg^−1^ h^−1^ and 22.53 mg N kg^−1^ h^−1^, with inhibition rates of 21.34% and 30.57%, respectively. The inhibition rates of NAR and NIR activities of the AgNPs treatment group were 44.19% and 65.03%. The NAR and NIR activities of the combined contaminated treatment groups were the lowest, with 16.84 mg N kg^−1^ h^−1^ and 6.96 mg N kg^−1^ h^−1^, and the inhibition rates were 53.80% and 72.76%, respectively. The NAR and NIR activities of three contaminated treatment groups showed significant differences compared to those of the control group (ANOVA, *p* < 0.05), and the inhibition effect on NIR activity was stronger than that on NAR activity (ANOVA, *p* < 0.05).

**Figure 7:**
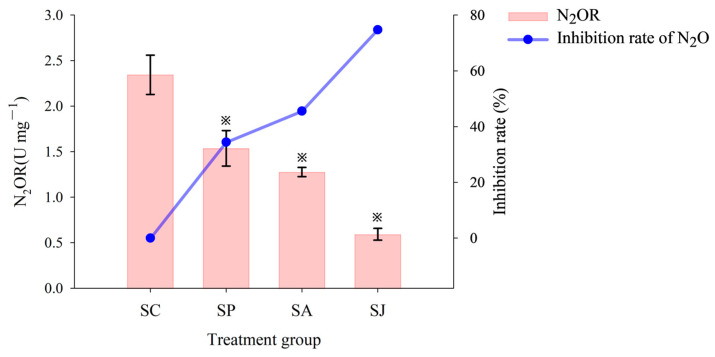
Activity of N_2_O reductase (N_2_OR) and inhibition rate of different treatment groups. “※” Represent the significant difference between group SC and the other three groups (ANOVA, *p* < 0.05).

The N_2_OR activity of sediments in different contaminated treatment groups are shown in Figure 7. The N_2_OR activity in the control group was the highest, which is 2.34 U mg^−1^. Followed by the Phe and AgNPs treatment groups, with N_2_OR activities were 1.54 U mg^−1^ and 1.28 U mg^−1^, and the inhibition rates were 35.44% and 45.58%, respectively. The N_2_OR activity of the combined contaminated treatment group was the lowest with 0.59 U mg^−1^, and the inhibition rate was 74.72%. The N_2_OR activities of three contaminated treatment groups exhibited significant differences compared to those of the control group (ANOVA, *p* < 0.05), and the inhibition effect on N_2_OR activity was stronger than that on NAR and NIR activities (ANOVA, *p* < 0.05). In general, NAR, NIR and N_2_OR activities were mostly inhibited by the combined contaminated treatment of Phe and AgNPs, followed by the AgNPs treatment, and the inhibition effect of Phe treatment was the least. However, the inhibition effect of three contaminated treatment groups on denitrification capacity (PDA, NO_3_^−^ and NO_2_^−^ reduction rates) varied in the order PDA > NO_3_^−^ reduction rate> NO_2_^−^ reduction rate. This may be related to the cascade effects of nutrients, pollution or enzymatic reactions, while reductases involved in denitrification were usually sequentially induced under anaerobic conditions [52].

Single and combined contamination of Phe and AgNPs had different degrees of inhibition effects on the PDA, NAR, NIR, N_2_OR, NO_3_^−^ and NO_2_^−^ reduction rates of sediment. Guo et al. (2011) [14] showed that the addition of PAHs (pyrene) could reduce PDA and denitrifying process-related reductase expression in a farmland soil. Some studies showed that PAHs can promote the increase in N_2_O accumulation while reducing the denitrification [53,54]. PAHs have the ability to diminish enzyme activity by disrupting the structure and composition of cell membranes and altering the conformation of protein molecules [55,56]. Moreover, the AgNPs could reduce the reduction rate of NO_3_^−^ under anaerobic conditions, and the reduction rate of NO_3_^−^ decreased significantly with the increase in AgNPs concentration [19,22]. The AgNPs entering the bacteria can attract each other with negatively charged biomacromolecules and change the three-dimensional conformation of the enzyme or replace the metal ions in the enzyme through binding with important functional groups such as oxygen, nitrogen and sulfhydryl groups of the enzyme, thus affecting the stability of the enzyme and catalytic activity of the enzyme [57,58]. AgNPs can also disrupt the integrity of the cell membrane, leading to the inactivation of membrane-bound enzymes and proteins [59]. In addition, AgNPs in the environment can release some dissolved silver ions (Ag^+^), which gradually approach and gather around bacteria under the action of electrostatic attraction; meanwhile, AgNPs and Ag^+^ can cause the inactivation of NIR by causing a three-dimensional conformation change [60]. NAR can be classified into two types—membrane-bound nitrate reductase (NAR) and periplasmic nitrate reductase (NAP)—and both NIR and N_2_OR are NAPs [61]. Therefore, NIR and N_2_OR, as NAPs, may be more sensitive to AgNPs. The combination of AgNPs and hydrocarbons could reduce microbial enzyme activity in estuary-mouth sediments [62]. Moreover, a previous study demonstrated that the combined contamination of AgNPs and phenol had a stronger inhibition effect on NO_3_^−^ and NO_2_^−^ reductase activities and NO_3_^−^ and NO_2_^−^ reducing ability than its single contamination [60]. In this study, the combination of Phe and AgNPs had a stronger inhibitory effect on denitrification than its single combination. A possible explanation is the toxic narcosis hypothesis proposed by Sikkema et al. (1994) [63], which states that the interaction between PAHs and lipophilic compounds on bacterial cell membranes can affect the integrity and permeability of cell membranes. Then, the activity of denitrifying bacteria is affected, leading to the inhibition of denitrification process.

### 3.3. Effect of Single and Combined Contamination of Phe and AgNPs on Total Bacterial Count and Relative Abundance of Denitrifying Genes

**Figure 8:**
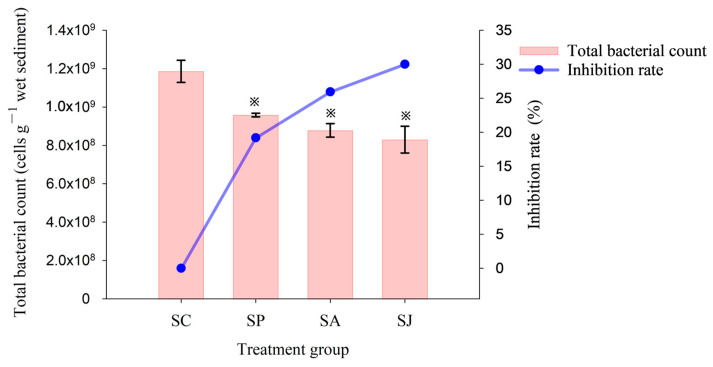
Total bacterial count and inhibition rate of different treatment groups. “※” Represent the significant difference between group SC and the other three groups (ANOVA, *p* < 0.05).

The short-term effects of single and combined contamination of Phe and AgNPs on the total bacteria count in the sediment of the JZB is shown in Figure 8. The total bacteria count in the control group was the highest at 1.19 × 10^9^ cells g^−1^. This was followed by the Phe and AgNPs treatment groups, with 9.58 × 10^8^ cells g^−1^ and 8.78 × 10^8^ cells g^−1^ and inhibition rates of 19.19% and 25.95%, respectively. The combined treatment group exhibited the lowest total bacterial count at 8.30 × 10^8^ cells g^−1^, with an inhibition rate of 30.00%. The total bacterial count in three contaminated treatment groups was significantly different from that in the control group (ANOVA, *p* < 0.05). Therefore, the order of the inhibitory effect of each contaminated treatment on total bacterial count was as follows: combined treatment > AgNPs treatment > Phe treatment.

**Figure 9:**
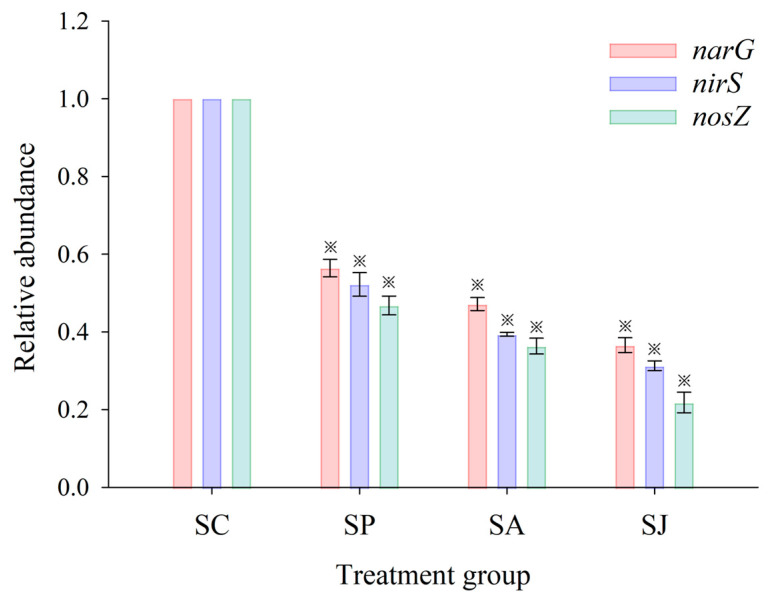
Relative abundances of denitrifying genes of different treatment groups. “※” Represent the significant difference between group SC and the other three groups (ANOVA, *p* < 0.05).

*narG*, *nirS and nosZ* genes play crucial roles in the denitrification process as they are responsible for the reduction of NO_3_^−^, NO_2_^−^ and N_2_O during denitrification, and their abundance directly affects the synthesis and function of related reductases. The relative abundances of the three genes in sediments of the three contaminated treatment groups are shown in Figure 9. The abundances of three denitrification functional genes in the three contaminated treatment groups were inhibited to varying degrees (ANOVA, *p* < 0.05). The *nosZ* gene exhibited the highest level of inhibition among the three denitrifying genes across all four treatment groups, with inhibition rates ranging from 53.23% to 78.19%; meanwhile, the *narG* gene was the least inhibited gene, and the inhibition rate ranged from 43.57% to 63.43%. The order of the inhibition effect of each contaminated treatment on the three denitrifying genes was as follows: *nosZ* > *nirS* > *narG*; the inhibition effect of the denitrifying genes was much larger than that of the total bacterial population (ANOVA, *p* < 0.05), which may indicate that contaminants may have some specific toxic effects on denitrifying bacteria rather the other bacteria. The *nosZ* gene of *Pseudomonas stutzeri* PCN-1 was more sensitive to heavy metal (Cd, Cu, Ni and Zn) contamination than the *nirS* gene under anaerobic conditions [64]. The inhibition effect of AgNPs treatment on the *nirS* gene was less than that on NO_2_^−^ reductase (ANOVA, *p* > 0.05), but the difference was not significant; the inhibition effect of AgNPs treatment on the *narG* and *nosZ* genes was significantly greater than that on NAR and N_2_OR (ANOVA, *p* < 0.05). This shows that the impact of short-term (6 days) AgNPs exposure on the NO_3_^−^ and N_2_O reduction processes of coastal sediment is mainly through the inhibition of the related functional genes rather than the reductase activity, while the impact of NO_2_^−^ reduction process may be through the combined inhibition of reductase activity and functional genes. Moreover, the inhibition effect of Phe treatment on *narG*, *nirS and nosZ* genes was significantly greater than that on NAR, NIR and N_2_OR (ANOVA, *p* < 0.05), which indicates that the impact of short-term (6 days) Phe exposure on the NO_3_^−^, NO_2_^−^ and N_2_O reduction processes of coastal sediment is mainly through the inhibition of the related functional genes rather than the reductase activity. The inhibition effect of the combined treatment on the *narG*, *nirS and nosZ* genes was slightly greater than that on NAR, NIR and N_2_OR (ANOVA, *p* > 0.05), and the difference was not significant. This indicates that the impact of the short-term (6 days) combined Phe and AgNPs exposure on NO_3_^−^, NO_2_^−^ and N_2_O reduction may occur through the combined inhibition of reductase activity and functional genes, but the toxic effect of denitrifying functional genes was slightly higher. In addition, correlation analysis of the *narG*, *nirS and nosZ* genes and corresponding reductases showed that there was a significant linear relationship between functional genes and their corresponding reductase activities (ANOVA, *p* < 0.05).

Our previous research demonstrated that the inhibition effect of AgNPs on total bacterial count and denitrifying genes abundance was less than that of Phe under acute (24 h) conditions (ANOVA, *p* > 0.05) [27,28]. However, the inhibition effect of three contaminated treatment groups on total bacterial count and relative abundance of denitrifying genes varied in the order combined treatment > AgNPs treatment > Phe treatment. The results obtained from this study suggest that the toxic effects of Phe and AgNPs on the total bacterial count and denitrifying genes abundance were different under acute and short-term contamination conditions. On one hand, it is based on the fact that the longer the AgNPs prevails in the environment, the higher the Ag^+^ content released and the stronger the toxic effect produced. Bao et al. (2016) [65] found that after 4 and 7 days contaminated with 35 nm, 75 nm and PVP-coated AgNPs in two freshwater sediments, the total bacterial count in AgNPs treatment groups decreased to varying degrees. On the other hand, it is due to some bacteria having the ability to utilize PAHs as their carbon and energy source, thereby promoting growth [66]. Some denitrifying bacteria can degrade PAHs and other hydrocarbons (aliphatic and aromatic hydrocarbons) [67,68]. In addition, some studies have shown that certain heavy metals, nanomaterials and PAHs can significantly reduce the abundance of denitrifying bacteria [69,70,71,72].

### 3.4. Community Diversity and Similarity of Denitrifying Bacteria after Single and Combined Exposure to Phe and AgNPs

#### 3.4.1. Alpha Diversity

Alpha diversity can be explained as the richness of a species community in the specific region, and it is mostly used to analyze the microbial community diversity within a sample. Diversity analysis of a single sample can reflect the richness and microbial community diversity within a sample and then evaluate the differences in the richness and diversity of the microbial communities among samples [73]. In this study, by comparing the microbial diversity indices (Shannon, Simpson, Chao1 and Ace) of different contaminated treatment groups, the differences of the diversity indices of *nirS* and *nosZ* genes in coastal sediment under single and combined short-term contamination conditions of Phe and AgNPs were studied. The OTU number and four diversity indices of the *nosZ* gene were higher than those of the *nirS* gene (Table S1). Both Chao1 and Ace indices serve as indicators of community richness, where higher values suggest a greater diversity of species within the community; the Shannon index and Simpson index are used to assess community diversity, with a higher Shannon index and a lower Simpson index indicating a more diverse community [74]. Single and combined contamination of Phe and AgNPs exhibited varying degrees of inhibition on the Shannon, Simpson, Chao1 and Ace indices (Table 1). Previous research showed that adding heavy metals or organic pollutants to soil can reduce microbial diversity [75,76], and the addition of AgNPs could also reduce soil microbial diversity [77].

The Shannon, Chao1 and Ace indices of the *nosZ* gene in the three contaminated treatment groups were larger than those of the *nirS* gene, while the Simpson index was smaller than that of the *nirS* gene, indicating that the diversity and richness of the *nosZ* gene were greater than those of the *nirS* gene, which is consistent with the result of the PDA being greater than the NO_2_^−^ reduction rate. This finding was inconsistent with the research in Taihu Lake sediments, which showed that the diversity and richness of the *nirS* gene were greater than those of the *nosZ* gene [21]. Through the analysis of Shannon and Simpson indices of different contaminated treatment groups, the order of inhibitory effect on the *nirS* gene was Phe treatment > AgNPs treatment > combined treatment; the inhibition effect of the three contaminated treatment groups on *nosZ* gene varied in the order AgNPs treatment > Phe treatment > combined treatment. This was inconsistent with the order of inhibitory effects on the relative abundance of the *nirS* and *nosZ* genes in different contaminated treatment groups. Through the analysis of Chao1 and Ace indices, which reflect community richness, we found that the order of inhibitory effect on the *nirS* and *nosZ* genes was Phe treatment > combined treatment > AgNPs treatment, which was also inconsistent with the inhibition order of the relative abundance of the *nirS* and *nosZ* genes in different contaminated treatments. The results suggested that there was no obvious correlation between the diversity of denitrifying bacterial communities and denitrification function. However, it was found that the denitrifying genes abundance was strongly associated with denitrification function. The denitrifying genes abundance could better predict the effects of PAHs and AgNPs stress on sediment denitrification than diversity. Earlier studies have proven that the activities of nitrifiers and denitrifying bacteria in soil were not affected by the reduction of microbial diversity under stable environmental conditions [78,79]. Guo et al. (2011) [14] also discovered that there was no substantial correlation between the PDA and the diversity of denitrifying communities, but there was a close relationship between the denitrification function and denitrifying bacterial abundance.

#### 3.4.2. Beta Diversity

**Figure 10:**
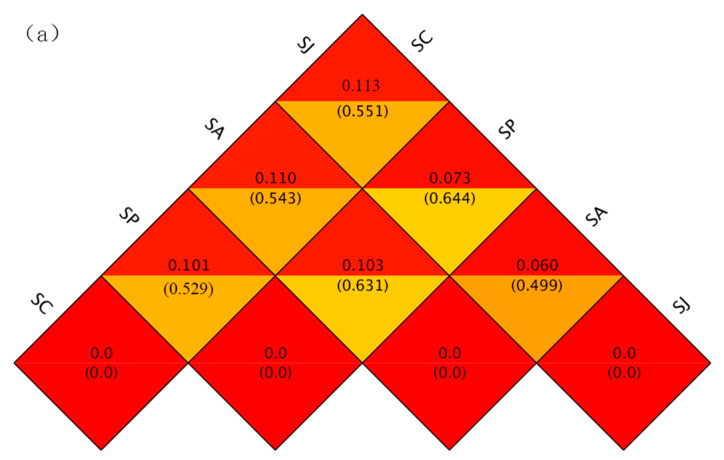

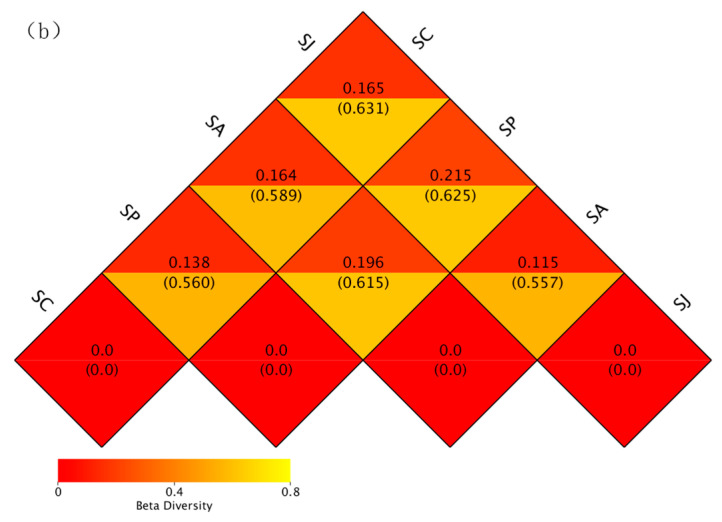
Thermal map of beta diversity index of *nosS* (**a**)- and *nosZ* (**b**)-type denitrifying bacteria between diffrernt groups. Note: in the same square, the upper and lower values represent weighted UniFrac and unweighted UniFrac distance, respectively.

Beta diversity can be defined as the magnitude of community composition differences between individual samples [79,80]. The weighted and unweighted UniFrac were proposed in 2005 and 2007, respectively; the former only considers the presence or absence of species, while the latter considers changes in species abundance. The UniFrac value is inversely proportional to the differences in species diversity between the samples [81,82]. The weighted and unweighted UniFrac distances were calculated between the control groups and three contaminated treatment groups based on the *nirS* and *nosZ* genes. The analysis revealed that the largest distances were observed between the control group and the combined treatment group. Specifically, the distances for the *nirS* gene were 0.113 (weighted) and 0.551 (unweighted), while for the *nosZ* gene, the distances were 0.165 (weighted) and 0.631 (unweighted). This was followed by the control group and the AgNPs treatment group; the weighted and unweighted UniFrac distances between the control group and the Phe treatment group were the smallest (Figure 10). The difference between the community diversity of the two genes of the contaminated treatment groups and the control group varied in the order combined treatment > AgNPs treatment > Phe treatment. In addition, the weighted and unweighted UniFrac distances of the two genes between the combined treatment group and the Phe treatment group were higher than those of the AgNPs treatment group, indicating that the difference in community diversity of the two genes between the combined treatment group and the AgNPs treatment group was smaller than that of the Phe treatment group.

#### 3.4.3. UPGMA Cluster Analysis

**Figure 11:**
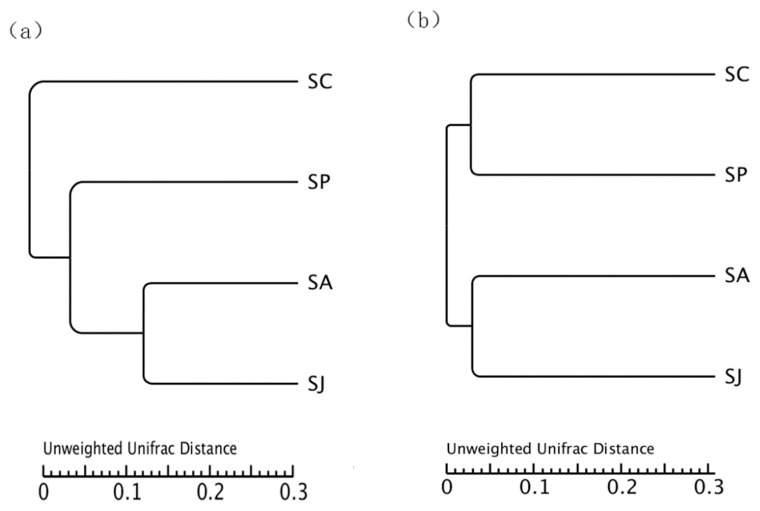
The UPGMA cluster analysis of *nosS* (**a**)- and *nosZ* (**b**)-type denitrifying bacteria based on unweighted UniFrac between diffrernt groups.

UPGMA cluster analysis was conducted on the groups based on the weighted UniFrac distance matrix (Figure 11). For the *nirS* gene, the similarity between the AgNPs and combined treatment groups was greater than that between the Phe and combined treatment groups. For the *nirS* and *nosZ* genes, the similarity between the AgNPs and combined treatment group was large, and these two groups had little similarity with the control and Phe treatment groups, which was consistent with the results of the beta diversity. The differences demonstrated by UPGMA cluster analysis among different treatment groups might be due to the different toxic effects of Phe, AgNPs and combined contamination on denitrifying bacteria with the *nirS* and *nosZ* genes.

### 3.5. Community Structure of Denitrifying Bacteria after Single and Combined Contamination of Phenanthrene and AgNPs

#### 3.5.1. Changes in Bacterial Community Structure at Phylum Level in Different Treatment Groups

**Figure 12:**
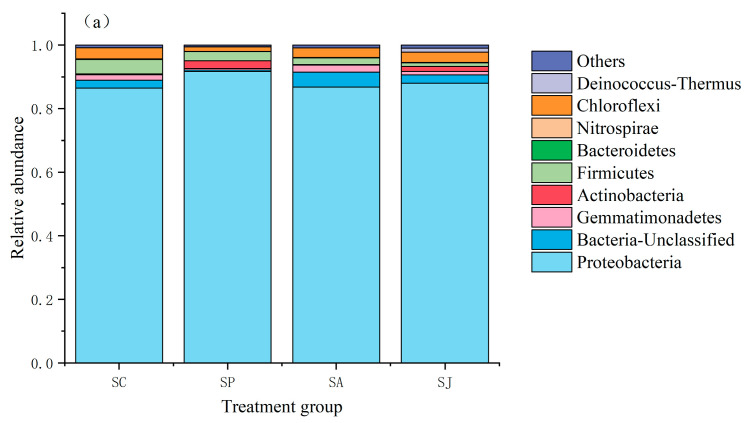

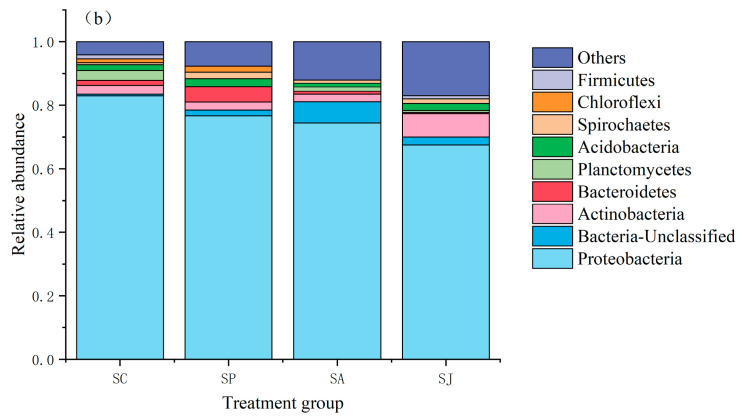
Community composition of *nosS* (**a**)- and *nosZ* (**b**)-type denitrifying bacteria at phylum level.

The denitrifying bacterial community composition of the *nirS* and *nosZ* genes at the phylum level is shown in Figure 12. There were 13 phyla, 18 classes, 35 orders, 45 families and 59 genera of *nirS*-type denitrifying bacteria. The *Proteobacteria* was the dominant bacteria in all treatment groups. The abundance ratios of *Proteobacteria* in the control group, Phe treatment group, AgNPs treatment group and combined treatment group were 86.51%, 91.78%, 86.87% and 88.00%, respectively. There was little difference between the untreated groups. In addition, there were 15 phyla, 25 classes, 58 orders, 80 families and 128 genera of *nosZ*-type denitrifying bacteria. *Proteobacteria* was also the dominant bacteria in all treatment groups. The abundance ratio of *Proteobacteria* in the control group was the highest (82.89%), followed by the Phe and AgNPs groups (76.66% and 74.39%), and the combined treatment group was the lowest (67.48%). The dominance of *Proteobacteria* in *nosZ*-type denitrifying bacteria was lower than that in *nirS*-type denitrifying bacteria, indicating that *Proteobacteria* of the *nosZ* gene were more sensitive to the added contaminations than those of the *nirS* gene. A previous study found that *Pseudomonadales*, *Burkholderiales*, *Rhizobiales* and *Rhodospirillale* belonging to *Proteobacteria* can degrade PAHs [14]. For *nirS*-type denitrifying bacteria, single and combined contamination of Phe and AgNPs promoted the dominance of *Actinobacteria* to varying degrees but inhibited the dominance of *Firmicutes* and *Chloroflexi* to a certain extent. Furthermore, the contamination with single AgNPs and combined Phe and AgNPs could promote the dominance of *Gemmatimonadetes*. For *nosZ*-type denitrifying bacteria, single and combined contamination with Phe and AgNPs had a certain inhibitory effect on the dominance of *Planctomycetes* and *Bacteroidetes* but had a promoting effect on the dominance of *Acidobacteria*. The single contamination with Phe and AgNPs had a weak inhibitory effect on the dominance of *Actinomycetota*, while the combined contamination with Phe and AgNPs had a certain promoting effect on it. Researchers have reported that *Proteobacteria*, *Actinobacteria* and *Acidobacteria* are commonly dominant phyla in soils contaminated by heavy metals, and these bacteria played an important role in the bioremediation of contaminated soils [83,84,85]. Denitrifying bacteria are mostly facultative anaerobic heterotrophic bacteria, including *Proteobacteria*, *Aquificae*, *Deinococcus-Thermus*, *Actinobacteria*, *Bacteroidetes* and *Firmicutes*, among which the *Pseudomonas* and *Bacillus* are ubiquitous in marine ecosystems. In addition, the denitrifying bacteria existed as *Actinobacteria*, *Chloroflexi*, *Bacteroidetes* and *Nitrospirae* [86]. Fan et al. [87] showed that the main groups of denitrifying bacteria in Liaohe River estuary sediments were *Proteobacteria*, *Firmicutes* and *Actinobacteria*, and *Proteobacteria* were the dominant group; these results are similar to the conclusions of this study.

#### 3.5.2. Changes in Bacterial Community Structure at the Genus Level in Different Treatment Groups

**Figure 13:**
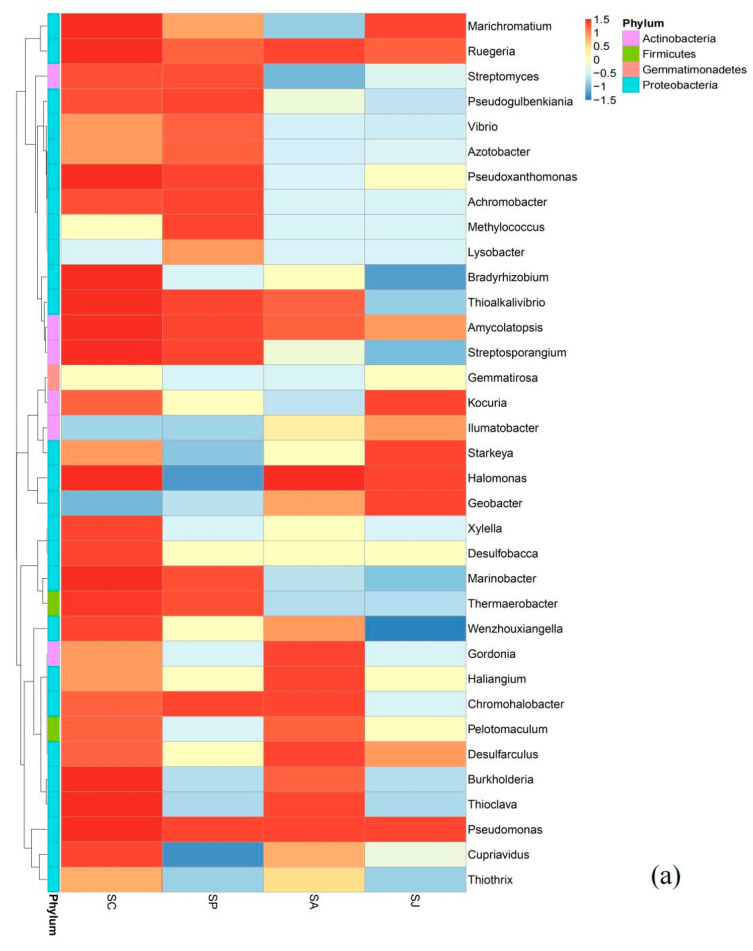

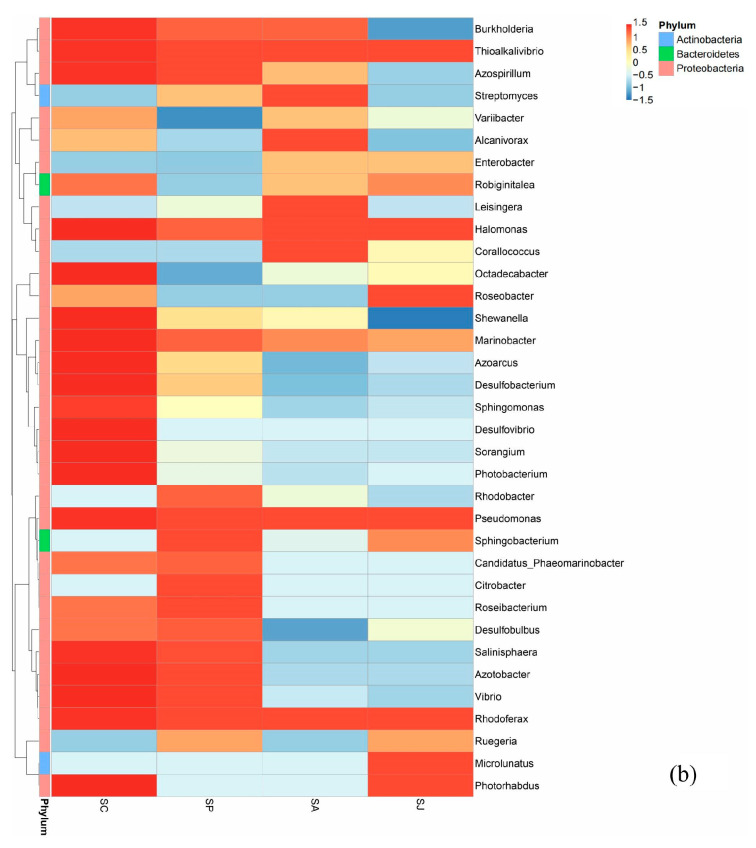
Heatmap graph of *nosS* (**a**)- and *nosZ* (**b**)-type denitrifying bacteria at genus level. Note: bluer color blocks indicate lower abundance, while redder color blocks signify higher abundance.

The heatmaps of the top 35 genera of *nosS*- and *nosZ*-type denitrifying bacteria are shown in Figure 13. The heatmap visually represents the data, where each color block signifies the bacterial community structure at the genus level in the different treatment groups. The intensity of color reflects the degree of similarity or difference between these groups.

AgNPs exposure can modify the microbial community composition of microorganisms in terrestrial, aquatic, marine and wastewater environments [88,89,90]. PAHs with different rings could also alter the community structure of soil microorganisms [91]. For *nosS*-type denitrifying bacteria, *Pseudomonas* was the most dominant in all treatment groups, and the abundance of *Pseudomonas* in the three contaminated treatment groups was obviously lower than that in the control group, but its abundance ratio increased. The abundance ratio of *Pseudomonas* in the control group was 27.53%, and the abundance ratios of *Pseudomonas* in the Phe, AgNPs and combined treatment groups were 40.45%, 37.45% and 43.28%, respectively. A previous study showed that the clonal proportion of *Pseudomonas* slightly increased before and after PAHs addition in a farmland soil [14]. In addition, the abundance and abundance ratio of *Bradyrhizobium* in all contaminated treatment groups were obviously lower than those in the control group. The abundance and abundance ratio of *Ruthenia* in both the Phe and combined contaminated treatment groups decreased, while the abundance in the AgNPs treatment group decreased and the abundance ratio increased. Moreover, the abundance and abundance ratio of *Chromohalobacter* in the Phe and combined contaminated treatment groups increased noticeably. Some scholars studied *nosS*-type denitrifying bacteria in farmland soil and found that the addition of PAHs could significantly increase the clonal proportion of *Pseudomonas* (*p* < 0.05) and reduce the clonal proportion of *Bradyrhizobium* and *Rhodobacteraceae* [14], which was consistent with this study whereby the abundance ratio of *Rugiella* and *Thioclava* belonging to *Rhodobacteraceae* in the phenanthrene treatment group decreased noticeably. This was also demonstrated in a PAHs-contaminated soil from an oil field, which showed that *Pseudomonas* and *Bradyrhizobium* of *nosS*-type denitrifying bacteria had a high proportion of cloned sequences [15]. In addition, the addition of AgNPs could also increase the abundance ratio of *Pseudomonas* among *nosS*-type denitrifying bacteria in a lake sediment [21]. For *nosZ*-type denitrifying bacteria, *Pseudomonas* was also the most dominant in all treatment groups, and the abundance of *Pseudomonas* in all three contaminated treatment groups was obviously lower than that in the control group, but its abundance ratio increased. The abundance ratio of *Pseudomonas* in the control group was 21.05%, and the abundance ratios of *Pseudomonas* in the Phe, AgNPs and combined treatment groups were 29.18%, 28.32% and 35.57%, respectively. Compared to the control group, the abundance and abundance ratio of *Burkholderia* and *Variibacter* in Phe treatment group were obviously lower, while *Sphingomonas* and *Azospirillum* were higher. This is due to the ability of *Sphingomonas* to degrade PAHs [92]. A previous study also demonstrated that the AgNPs can increase the abundance ratio of *Pseudomonas* among *nosZ*-type denitrifying bacteria in a lake sediment [21]. In short, both Phe and AgNPs contamination can increase the abundance ratio of *Pseudomonas* among *nosS*- and *nosZ*-type denitrifying bacteria in coastal marine sediment, and the promotion effect of combined contamination with Phe and AgNPs on *Pseudomonas*, rather than the other bacteria, was stronger than their single contamination.

### 3.6. Environmental Implications

Our study indicated that single and combined contamination with Phe and AgNPs could inhibit denitrification activity, denitrifying enzyme activity, relative abundance of denitrifying genes and denitrifying bacterial diversity; meanwhile, it could also change denitrifying bacterial diversity structure and further proceed to change the nitrogen-transforming rates, boosting the nitrogen loss in the water–sediment systems of a coastal area. Furthermore, the inhibition effect of combined contamination with Phe and AgNPs on denitrification and denitrifying bacteria was stronger than single contamination.

Although our research has provided valuable insights into assessing the single and combined effects of Phe and AgNPs on denitrification in coastal environment, further investigations are essential to form a comprehensive understanding. The toxicity and bioavailability of PAHs and AgNPs in the natural environment have been shown to closely correlate with their physicochemical properties, such as the ring content of PAHs and the size and coating of AgNPs [65,74,93]. Additionally, the antibacterial properties of PAHs and AgNPs may be significantly influenced by environmental factors, including pH, dissolved oxygen levels and organic matter content [94,95]. Moreover, different bacterial species with similar functions but inhabiting distinct environments may exhibit varying sensitivities to PAHs and AgNPs [96,97]. Thus, the consequences of Phe and AgNPs on denitrification may vary among different coastal areas, and further explorations are needed.

## 4. Conclusions

Based on the experimental results, all contaminated treatment groups had different degrees of inhibitory effect on denitrification activity, denitrifying enzyme activity, total bacteria count and relative abundance of denitrifying genes. The inhibitory effect sequence of each treatment group was combined contamination > AgNPs contamination > Phe contamination. Moreover, the inhibitory effects of denitrifying genes were much larger than that of total bacteria count, indicating that the pollutants had specific toxic effects on denitrifying bacteria. The sequence of sensitivity of three reduction process to contaminations was N_2_O > NO_2_^−^ > NO_3_^−^. All contaminated treatment groups could increase NO_3_^−^, NO_2_^−^ and N_2_O accumulation. Furthermore, there was a significant linear relationship between functional gene abundance and reductase activity. The inhibitory effect of AgNPs on NO_3_^−^ and N_2_O reduction was mainly through the inhibition of gene function, while the NO_2_^−^ reduction was through the inhibition of both reductase activity and gene function. The inhibitory effect of Phe on NO_3_^−^, NO_2_^−^ and N_2_O reduction was mainly through the inhibition of gene function. The inhibitory effect of the combined contamination on NO_3_^−^, NO_2_^−^ and N_2_O reduction was through the inhibition of both reductase activity and gene function. Furthermore, it was found that denitrifying genes abundance could better predict the influence of Phe and AgNPs on sediment denitrification than the diversity of denitrifying bacteria. In addition, the community structure of *nosS*- and *nosZ*-type denitrifying bacteria changed dramatically at the genus level but not greatly at the phylum level. Single and combined contamination with Phe and AgNPs can reduce the dominance of *Pseudomonas*, which may slow down the degradation of PAHs and inhibit the denitrification, especially the combined pollution.

Thus, our results strongly indicate that both single and combined contamination of Phe and AgNPs have a notable suppressive effect on denitrification and denitrifying bacterial communities in a typical coastal marine sediment, with a combined contamination of Phe and AgNPs posing an increased threat to denitrifying bacteria in the short term. These results further imply that coastal areas like the JZB could be facing a toxicity risk from Phe and AgNPs all around the world. Compared with a single exposure, using repeated exposure of pollutants to investigate dynamic processes of microbial activity and function is more indicative for assessing the ecological risks of pollutants in the real environment. To sum up, although the impacts of Phe and AgNPs are notable, their concentrations in coastal areas remain lower than the exposure concentration in this study. The findings underscore the importance of maintaining the current environmental status. In addition, this study also provides a basis for the ecological security and health risk assessment of single and combined contamination of Phe and AgNPs in coastal marine sediment.

## Figures and Tables

**Table 1:** Different microbial diversity indices of *nosS*- and *nosZ*-type denitrifying bacteria in different treatment groups.

Gene	Sample	OTUs	Shannon	Simpson	Chao1	Ace	Coverage
*nirS*	SC	454	2.54	0.309	509	519	0.999
	SP	405	1.60	0.680	409	425	0.999
	SA	442	1.89	0.637	501	508	0.999
	SJ	399	2.28	0.552	448	460	0.999
*nosZ*	SC	1038	3.39	0.774	1962	1538	0.996
	SP	964	3.14	0.793	873	931	0.995
	SA	836	2.98	0.837	1195	1239	0.997
	SJ	721	3.25	0.776	1064	1009	0.997

## Data Availability

Data are contained within the article and Supplementary Materials.

## Supplementary Materials

The following supporting information can be downloaded at: https://www.mdpi.com/article/10.3390/microorganisms12040745/s1, Table S1: Quality control of data among different treatment groups.

## References

[1] Hsiao S.Y., Hsu T.C., Liu J.W., Xie X., Zhang Y., Lin J., Wang H., Yang J.Y., Hsu S.C., Dai M. (2014). Nitrification and its oxygen consumption along the turbid Chang Jiang River plume. Biogeosciences.

[2] Rabalais N.N. (2002). Nitrogen in aquatic ecosystems. Ambio.

[3] Jia Z., Liu T., Xia X., Xia N. (2016). Effect of particle size and composition of suspended sediment on denitrification in river water. Sci. Total Environ..

[4] Harter J., Weigold P., El-Hadidi M., Huson D.H., Kappler A., Behrens S. (2016). Soil biochar amendment shapes the composition of N_2_O-reducing microbial communities. Sci. Total Environ..

[5] Crutzen P.J. (1970). The influence of nitrogen oxides on the atmospheric ozone content. Q. J. R. Meteorol. Soc..

[6] Dickinson R.E., Cicerone R.J. (1986). Future global warming from atmospheric trace gases. Nature..

[7] Backhaus T., Blanck H., Faust M. (2010). Hazard and Risk Assessment of Chemical Mixtures under REACH: State of the Art, Gaps and Options for Improvement.

[8] McGillicuddy E., Murray I., Kavanagh S., Morrison L., Fogarty A., Cormican M., Dockery P., Prendergast M., Rowan N., Morris D. (2017). Silver nanoparticles in the environment: Sources, detection and ecotoxicology. Sci. Total Environ..

[9] Verma P., Maheshwari S.K. (2019). Applications of silver nanoparticles in diverse sectors. Int. J. Nano Dimens..

[10] Gottschalk F., Sonderer T., Scholz R.W., Nowack B. (2009). Modeled environmental concentrations of engineered nanomaterials (TiO_2_, ZnO, Ag, CNT, fullerenes) for different regions. Environ. Sci. Technol..

[11] Griffitt R.J., Hyndman K., Denslow N.D., Barber D.S. (2009). Comparison of molecular and histological changes in zebrafish gills exposed to metallic nanoparticles. Toxicol Sci..

[12] Wang X., Yuan K., Chen B., Lin L., Huang B., Luan T. (2016). Monthly variation and vertical distribution of parent and alkyl polycyclic aromatic hydrocarbons in estuarine water column: Role of suspended particulate matter. Environ. Pollut..

[13] Cao C., Huang J., Yan C.N., Zhang X.X., Ma Y.X. (2021). Impacts of Ag and Ag_2_S nanoparticles on the nitrogen removal within vertical flow constructed wetlands treating secondary effluent. Sci. Total Environ..

[14] Guo G.X., Deng H., Qiao M., Mu Y.J., Zhu Y.G. (2011). Effect of pyrene on denitrification activity and abundance and composition of denitrifying community in an agricultural soil. Environ. Pollut..

[15] Yao Y.H., Wang M.X., Zuo X.H., Li Z.L., Luo F., Zhou Z.F. (2016). Effects of PAHs Pollution on the Community Structure of Denitrifiers in a Typical Oilfield. Huanjing Kexue/Environ. Sci..

[16] Zhou Z.F., Yao Y.H., Wang M.X., Zuo X.H. (2017). Co-effects of pyrene and nitrate on the activity and abundance of soil denitrifiers under anaerobic condition. Arch. Microbiol..

[17] Wuebbles D. (2009). Nitrous oxide: No laughing matter. Science.

[18] Jin B., Liu Y., Li X., Hou J., Bai Z., Wang L., Zhao J. (2022). New insights into denitrification and phosphorus removal with degradation of polycyclic aromatic hydrocarbons in two-sludge system. Bioresour. Technol..

[19] VandeVoort A.R., Skipper H., Arai Y. (2014). Macroscopic assessment of nanosilver toxicity to soil denitrification kinetics. J. Environ. Qual..

[20] Zheng Y., Hou L., Liu M., Newell S.E., Yin G., Yu C., Zhang H., Li X., Gao D., Gao J. (2017). Effects of silver nanoparticles on nitrification and associated nitrous oxide production in aquatic environments. Sci. Adv..

[21] Liu S., Wang C., Hou J., Wang P., Miao L., Fan X., You G., Xu Y. (2018). Effects of Ag and Ag_2_S nanoparticles on denitrification in sediments. Water Res..

[22] VandeVoort A.R., Arai Y. (2012). Effect of silver nanoparticles on soil denitrification kinetics. Ind. Biotechnol..

[23] Xu J., Bao S.P., Xiang D.F., Xue L., Tang W., Fang T. (2023). Effects of silver nanoparticles on denitrification and anammox in sediments of hypertrophic and mesotrophic lakes. Sci. Total Environ..

[24] Liu S., Miao L.Z., Li B.L., Shan S.J., Li D.P., Hou J. (2023). Long-term effects of Ag NPs on denitrification in sediment: Importance of Ag NPs exposure ways in aquatic ecosystems. Water Res..

[25] Dai Z.X., Yin Y., Wang S.H. (2013). Effect of nanomaterials on ecotoxicity of phenanthrene in Carassius auratus. Environ. Chem..

[26] Schwab F., Bucheli T.D., Camenzuli L., Magrez A., Knauer K., Sigg L., Nowack B. (2013). Diuron sorbed to carbon nanotubes exhibits enhanced toxicity to Chlorella vulgaris. Environ. Sci. Technol..

[27] Sun P.F., Bai J., Li K.R., Zhao Y.G., Tian Y.Z. (2020). Impacts of phenanthrene on denitrification activity and transcription of related functional genes in estuarine and marine sediments. J. Ocean Univ. China..

[28] Sun P.F., Li K.R., Yi S.K., Li H., Chen X. (2022). Effects of silver nanoparticles on denitrification and associated N_2_O release in estuarine and marine sediments. J. Ocean Univ. China..

[29] Wigginton N.S., Haus K.L., Hochella M.F. (2007). Aquatic environmental nanoparticles. J. Environ. Monit..

[30] Ren J.X., Ma Q.M., Yang X.N., Li Y.J. (2017). Characteristic parameters of organic hydrocarbons in the surface sediments of Jiaozhou bay. Mar. Environ. Res..

[31] Fang H., Liang H., Guosen Z. (2018). Distribution and Sources of Polycyclic Aromatic Hydrocarbons in Surface Sediments from the East China Sea. Earth Environ..

[32] Zhang Z., Lo I.M.C., Zheng G., Woon K.S., Rao P. (2015). Effect of autotrophic denitrification on nitrate migration in sulfide-rich marine sediments. J. Soils Sediments..

[33] Sørensen J. (1978). Denitrification rates in a marine sediment as measured by the acetylene inhibition technique. Appl. Environ. Microbiol..

[34] Joye S.B., Smith S.V., Hollibaugh J.T., Paerl H.W. (1996). Estimating denitrification rates in estuarine sediments: A comparison of stoichiometric and acetylene based methods. Biogeochemistry.

[35] Wigginton N.S., de Titta A., Piccapietra F., Dobias J., Nesatyy V.J., Suter M.J.F., Bernier-Latmani R. (2010). Binding of silver nanoparticles to bacterial proteins depends on surface modifications and inhibits enzymatic activity. Environ. Sci. Technol..

[36] Schug H., Isaacson C.W., Sigg L., Ammann A.A., Schirmer K. (2014). Effect of TiO_2_ nanoparticles and uv radiation on extracellular enzyme activity of intact heterotrophic biofilms. Environ. Sci. Technol..

[37] Kristjansson J.K., Hollocher T.C. (1980). First practical assay for soluble nitrous oxide reductase of denitrifying bacteria and a partial kinetic characterization. J. Biol. Chem..

[38] Zhu X., Chen Y. (2011). Reduction of N_2_O and NO generation in anaerobic−aerobic (low dissolved oxygen) biological wastewater treatment process by using sludge alkaline fermentation liquid. Environ. Sci. Technol..

[39] López-Gutiérrez J.C., Henry S., Hallet S., Martin-Laurent F., Catroux G., Philippot L. (2004). Quantification of a novel group of nitrate-reducing bacteria in the environment by real-time PCR. J. Microbiol. Methods..

[40] Hammes F.A., Egli T. (2005). New method for assimilable organic carbon determination using flow-cytometric enumeration and a natural microbial consortium as inoculum. Environ. Sci. Technol..

[41] Throbäck I.N., Enwall K., Jarvis Å., Hallin S. (2004). Reassessing PCR primers targeting *nirS*, *nirK* and *nosZ* genes for community surveys of denitrifying bacteria with DGGE. FEMS Microbiol. Ecol..

[42] Henry S., Bru D., Stres B., Hallet S., Philippot L. (2006). Quantitative detection of the *nosZ* gene, encoding nitrous oxide reductase, and comparison of the abundances of 16S rRNA, *narG*, *nirK*, and *nosZ* genes in soils. Appl. Environ. Microbiol..

[43] Livak K.J., Schmittgen T.D. (2001). Analysis of Relative Gene Expression Data Using Real-Time Quantitative PCR and the 2^−ΔΔCT^ Method. Methods..

[44] Huang X., Lee P.H. (2021). Shortcut nitrification/denitrification through limited-oxygen supply with two extreme COD/N-and-ammonia active landfill leachates. Chen. En. J..

[45] Magoč T., Salzberg S.L. (2011). FLASH: Fast length adjustment of short reads to improve genome assemblies. Bioinformatics..

[46] Edgar R.C. (2013). UPARSE: Highly accurate OTU sequences from microbial amplicon reads. Nat. Methods..

[47] Caporaso J.G., Kuczynski J., Stombaugh J., Bittinger K., Bushman F.D., Costello E.K., Fierer N., Peña A.G., Goodrich J.K., Gordon J.I. (2010). QIIME allows analysis of high-throughput community sequencing data. Nat. Methods..

[48] Wang Q., Garrity G.M., Tiedje J.M., Cole J.R. (2007). Naive Bayesian classifier for rapid assignment of rRNA sequences into the new bacterial taxonomy. Appl. Environ. Microbiol..

[49] Zhong J., Fan C., Liu G., Zhang L., Shang J., Gu X. (2010). Seasonal variation of potential denitrification rates of surface sediment from Meiliang Bay, Taihu Lake, China. J. Environ. Sci-China..

[50] Magalhães C.M., Machado A., Matos P., Bordalo A.A. (2011). Impact of copper on the diversity, abundance and transcription of nitrite and nitrous oxide reductase genes in an urban European estuary. FEMS Microbiol. Ecol..

[51] Khalil M.A.K., Rasmussen R.A. (1988). Nitrous oxide: Trends and global mass balance over the last 3000 years. Ann. Glaciol..

[52] Philippot L., Hallin S. (2005). Finding the missing link between diversity and activity using denitrifying bacteria as a model functional community. Curr. Opin. Microbiol..

[53] Contreras-Ramos S.M., Alvarez-Bernal D., Montes-Molina J.A., Van Cleemput O., Dendooven L. (2009). Emission of nitrous oxide from hydrocarbon contaminated soil amended with waste water sludge and earthworms. Appl. Soil Ecol..

[54] Alburquerque J.A., Sánchez-Monedero M.A., Roig A., Cayuela M.L. (2015). High concentrations of polycyclic aromatic hydrocarbons (naphthalene, phenanthrene and pyrene) failed to explain biochar’s capacity to reduce soil nitrous oxide emissions. Environ. Pollut..

[55] Xue W., Warshawsky D. (2005). Metabolic activation of polycyclic and heterocyclic aromatic hydrocarbons and DNA damage: A review. Toxicol. Appl. Pharmacol..

[56] Yu H., Xia Q., Yan J., Herreno-Saenz D., Wu Y.S., Tang I.W., Fu P.P. (2006). Photoirradiation of polycyclic aromatic hydrocarbons with UVA light–a pathway leading to the generation of reactive oxygen species, lipid peroxidation, and DNA damage. Int. J. Environ. Res. Public Health..

[57] Sotiriou G.A., Pratsinis S.E. (2010). Antibacterial activity of nanosilver ions and particles. Environ. Sci. Technol..

[58] You C., Han C., Wang X., Zheng Y., Li Q., Hu X., Sun H. (2012). The progress of silver nanoparticles in the antibacterial mechanism, clinical application and cytotoxicity. Mol. Biol. Rep..

[59] Pal S., Tak Y.K., Song J.M. (2007). Does the antibacterial activity of silver nanoparticles depend on the shape of the nanoparticle? A study of the gram-negative bacterium Escherichia coli. Appl. Environ. Microbiol..

[60] Zou Q.Y. (2015). Composite Toxic Effects and Mechanisms of AgNPs and Phenol on Pure Denitrifying Bacteria. Master’s Thesis.

[61] Guo L.Y., Shi F., Yang L.Y. (2011). Advances in functional genes and molecular ecology in denitrifiers. Microbiol. China.

[62] Beddow J., Stölpe B., Cole P.A., Lead J.R., Sapp M., Lyons B.P., McKew B., Steinke M., Benyahia F., Colbeck I. (2014). Estuarine sediment hydrocarbon-degrading microbial communities demonstrate resilience to nanosilver. Int. Biodeter. Biodegr..

[63] Sikkema J., de Bont J.A., Poolman B. (1994). Interactions of cyclic hydrocarbons with biological membranes. J. Biol. Chem..

[64] Gui M., Chen Q., Ma T., Zheng M., Ni J. (2017). Effects of heavy metals on aerobic denitrification by strain *Pseudomonas stutzeri* PCN-1. Appl. Microbiol. Biotechnol..

[65] Bao S., Wang H., Zhang W.C., Xie Z.C., Fang T. (2016). An investigation into the effects of silver nanoparticles on natural microbial communities in two freshwater sediments. Environ. Pollut..

[66] Wilson S.C., Jones K.C. (1993). Bioremediation of soil contaminated with polynuclear aromatic hydrocarbons (PAHs): A review. Environ. Pollut.

[67] Yang X.H., Tang W.H., Li S.Y., Hong J. (2009). Isolation, identification and degradation ability of phenanthrene-degrading bacteria from contaminated soils. Res. Environ. Sci..

[68] Zhao B., Wang H., Li R., Mao X. (2010). *Thalassospira xianhensis* sp. nov., a polycyclic aromatic hydrocarbon-degrading marine bacterium. Int. J. Syst. Evol. Micr..

[69] Sobolev D., Begonia M. (2008). Effects of heavy metal contamination upon soil microbes: Lead-induced changes in general and denitrifying microbial communities as evidenced by molecular markers. Int. J. Environ. Res. Public Health.

[70] Wu Y.Y., Wu Q.H., Huang S., Ye J.X., Zhang H.J., Zhang R.D. (2012). Effect of polycyclic aromatic hydrocarbons on the vertical distribution of denitrifying genes in river sediments. Huan Jing Ke Xue.

[71] Zheng X., Su Y., Chen Y., Wan R., Liu K., Li M., Yin D. (2014). Zinc oxide nanoparticles cause inhibition of microbial denitrification by affecting transcriptional regulation and enzyme activity. Environ. Sci. Technol..

[72] Yu W., Liu R., Wang J., Xu F., Shen Z. (2015). Source apportionment of PAHs in surface sediments using positive matrix factorization combined with GIS for the estuarine area of the Yangtze River, China. Chemosphere.

[73] Li B., Zhang X., Guo F., Wu W., Zhang T. (2013). Characterization of tetracycline resistant bacterial community in saline activated sludge using batch stress incubation with high-throughput sequencing analysis. Water Res..

[74] Zhang C., Hu Z., Li P., Gajaraj S. (2016). Governing factors affecting the impacts of silver nanoparticles on wastewater treatment. Sci. Total Environ..

[75] Bamborough L., Cummings S.P. (2009). The impact of increasing heavy metal stress on the diversity and structure of the bacterial and actinobacterial communities of metallophytic grassland soil. Biol. Fertil. Soils..

[76] Xie Y., Fan J., Zhu W., Amombo E., Chen L. (2016). Effect of heavy metals pollution on soil microbial diversity and bermudagrass genetic variation. Front. Plant Sci..

[77] Samarajeewa A.D., Velicogna J.R., Princz J.I., Subasinghe R.M., Scroggins R.P., Beaudette L.A. (2017). Effect of silver nano-particles on soil microbial growth, activity and community diversity in a sandy loam soil. Environ. Pollut..

[78] Wertz S., Degrange V., Prosser J.I., Poly F., Commeaux C., Freitag T., Guillaumaud N., Le Roux X. (2006). Maintenance of soil functioning following erosion of microbial diversity. Environ. Microbiol..

[79] Rosenzweig M.L. (1995). Species Diversity in Space and Time.

[80] Legendre P., De Cáceres M. (2013). Beta diversity as the variance of community data: Dissimilarity coefficients and partitioning. Ecol. Lett..

[81] Lozupone C., Knight R. (2005). UniFrac: A new phylogenetic method for comparing microbial communities. Appl. Environ. Microbiol..

[82] Lozupone C.A., Hamady M., Kelley S.T., Knight R. (2007). Quantitative and qualitative β diversity measures lead to different insights into factors that structure microbial communities. Appl. Environ. Microbiol..

[83] Idris R., Trifonova R., Puschenreiter M., Wenzel W.W., Sessitsch A. (2004). Bacterial communities associated with flowering plants of the Ni hyperaccumulator *Thlaspi goesingense*. Appl Env. Microbiol..

[84] Spain A.M., Krumholz L.R., Elshahed M.S. (2009). Abundance, composition, diversity and novelty of soil Proteobacteria. ISME J..

[85] Azarbad H., Niklinska M., Laskowski R., van Straalen N.M., van Gestel C.A., Zhou J., He Z., Wen C., Röling W.F. (2015). Microbial community composition and functions are resilient to metal pollution along two forest soil gradients. FEMS Microbiol Ecol..

[86] Wei G., Li M., Li F., Li H., Gao Z. (2016). Distinct distribution patterns of prokaryotes between sediment and water in the Yellow River estuary. Appl. Microbiol. Biotechnol..

[87] Fan J.F., Chen J.Y., Chen G.L., Guan D.M. (2011). Study on the Quantity and Diversity of Denitrifying Bacteria in Sediments of Liaohe Estuary. Acta Oceanol. Sin. Chin. Ed..

[88] Li W., Luo D., Adyel T.M., Wu J., Miao L., Kong M., Hou J. (2023). Dynamic responses of carbon metabolism of sediment microbial communities to Ag nanoparticles: Effects of the single and repeated exposure scenarios. Sci. Total Environ..

[89] Bao S., Xue L., Xiang D., Xian B., Tang W., Fang T. (2023). Silver nanoparticles alter the bacterial assembly and antibiotic resistome in biofilm during colonization. Environ. Science-Nano.

[90] Rees A.P., Faraggiana E., Tait K., Celussi M., Dafnomilli E., Manna V., Manning A., Pitta P., Tsiola A., Živanović S. (2022). The presence of silver nanoparticles reduces demand for dissolved phosphorus to the benefit of biological nitrogen fixation in the coastal eastern Mediterranean Sea. Front. Mar. Sci..

[91] Zhang H., Liu X., Wang Y., Duan L., Liu X., Zhang X., Dong L. (2023). Deep relationships between bacterial community and polycyclic aromatic hydrocarbons in soil profiles near typical coking plants. Environ. Sci. Pollut. R..

[92] Haritash A.K., Kaushik C.P. (2009). Biodegradation aspects of polycyclic aromatic hydrocarbons (PAHs): A review. J. Hazard. Mater..

[93] Krzyszczak A., Dybowski M.P., Zarzycki R., Kobyłecki R., Oleszczuk P., Czech B. (2022). Long-term physical and chemical aging of biochar affected the amount and bioavailability of PAHs and their derivatives. J. Hazard. Mater..

[94] Jin B., Niu J., Liu Y., Zhao J., Yin Z. (2020). Effects of polycyclic aromatic hydrocarbons on sludge performance for denitrification and phosphorus removal. Chem. Eng. J..

[95] Wang X., Chen Y.P., Liu S.Y., Guo J.S., Fang F., Yan P. (2023). The effect of silver nanoparticles on aerobic denitrifying bacteria during biological nitrogen removal: A new perspective based on morphological effects. Chem. Eng. J..

[96] Zhang Z.Z., Cheng Y.F., Xu L.Z.J., Bai Y.H., Jin R.C. (2018). Anammox granules show strong resistance to engineered silver nanoparticles during long-term exposure. Bioresour. Technol..

[97] Premnath N., Mohanrasu K., Rao R.G.R., Dinesh G.H., Prakash G.S., Ananthi V., Ponnuchamy K., Muthusamy G., Arun A.A. (2021). Crucial Review on Polycyclic Aromatic Hydrocarbons—Environmental Occurrence and Strategies for Microbial Degradation. Chemosphere.

