# Supplementary material for: Single and Combined Effects of Phenanthrene and Silver Nanoparticles on Denitrification Processes in Coastal Marine Sediments
Source: Microorganisms. 2024 Apr 6;12(4):745. doi: 10.3390/microorganisms12040745 (PMC11051833; doi:10.3390/microorganisms12040745)
Supplement: Supplementary file 1 [file microorganisms-12-00745-s001.zip › microorganisms-2948765-supplementary.pdf]

Table.S1 Quality control of data among different treatment groups

Gene	Sample	Raw PE	Effective Tags	AvgLen(nt)	Q20	Q30	GC%	Effective%
nirS	SC	90,179	82,940	370	98.48	86.77	61.86	91.97
	SP	84,316	78,044	370	98.48	86.78	62.28	92.56
	SA	88,275	81,545	370	98.52	86.86	62.41	92.38
	SJ	80,573	72,715	370	98.48	86.77	62.04	90.25
nosZ	SC	98,065	95,066	223	99.07	88.05	57.13	96.94
	SP	95,780	91,944	224	99.17	88.23	56.87	96.00
	SA	90,576	88,041	223	99.18	88.25	57.24	97.20
	SJ	89,848	85,504	223	99.31	88.52	56.57	95.17

Note: Raw PE refers to the original off-machine PE reads; Effective Tags refer to Tags sequences that are finally used for subsequent analysis after filtering chimeras; AvgLen refers to the average length of Effective Tags; Q20 and Q30 refer to the percentage of bases in Effective Tags with base quality values greater than 20 (sequencing error rate less than 1%) and 30 (sequencing error rate less than 0.1%); GC (%) represents the content of GC bases in Effective Tags; Effective (%) represents the percentage of the number of Effective Tags to the number of Raw PE.
